# Impact of Gamma Irradiation on the Properties of Magnesium-Doped Hydroxyapatite in Chitosan Matrix

**DOI:** 10.3390/ma15155372

**Published:** 2022-08-04

**Authors:** Daniela Predoi, Carmen Steluta Ciobanu, Simona Liliana Iconaru, Silviu Adrian Predoi, Mariana Carmen Chifiriuc, Steinar Raaen, Monica Luminita Badea, Krzysztof Rokosz

**Affiliations:** 1 National Institute of Materials Physics, Atomistilor Street, No. 405A, 77125 Magurele, Romania; 2 Département de Physique, École Normale Supérieure Paris-Saclay, 4 Avenue des Sciences, 91190 Gif-sur-Yvette, France; 3 Physique Fondamentale, Université Paris-Saclay, 3 Rue Joliot Curie, 911190 Gif-sur-Yvette, France; 4 Life, Environmental and Earth Sciences Division, Research Institute of the University of Bucharest (ICUB), University of Bucharest, 060023 Bucharest, Romania; 5 Academy of Romanian Scientists, 54 Spl. Independentei St., District 5, 050085 Bucharest, Romania; 6 Biological Sciences Division, The Romanian Academy, 25, Calea Victoriei, Sector 1, District 1, 010071 Bucharest, Romania; 7 Department of Physics, Norwegian University of Science and Technology (NTNU), Realfagbygget E3-124 Høgskoleringen 5, NO 7491 Trondheim, Norway; 8 Faculty of Horticulture, University of Agronomic Sciences and Veterinary Medicine, 59 Marasti Blvd., 011464 Bucharest, Romania; 9 Koszalin University of Technology, Śniadeckich 2, PL 75-453 Koszalin, Poland

**Keywords:** magnesium, hydroxyapatite, chitosan, gamma irradiation, antifungal activity

## Abstract

This is the first report regarding the effect of gamma irradiation on chitosan-coated magnesium-doped hydroxyapatite (x_Mg_ = 0.1; 10 MgHApCh) layers prepared by the spin-coating process. The stability of the resulting 10 MgHApCh gel suspension used to obtain the layers has been shown by ultrasound measurements. The presence of magnesium and the effect of the irradiation process on the studied samples were shown by X-ray photoelectron spectroscopy (XPS). The XPS results obtained for irradiated 10 MgHApCh layers suggested that the magnesium and calcium contained in the surface layer are from tricalcium phosphate (TCP; Ca_3_(PO_4_)_2_) and hydroxyapatite (HAp). The XPS analysis has also highlighted that the amount of TCP in the surface layer increased with the irradiation dose. The energy-dispersive X-ray spectroscopy (EDX) evaluation showed that the calcium decreases with the increase in the irradiation dose. In addition, a decrease in crystallinity and crystallite size was highlighted after irradiation. By atomic force microscopy (AFM) we have obtained images suggesting a good homogeneity of the surface of the non-irradiated and irradiated layers. The AFM results were also sustained by the scanning electron microscopy (SEM) images obtained for the studied samples. The effect of gamma-ray doses on the Fourier transform infrared spectroscopy (ATR-FTIR) spectra of 10 MgHApCh composite layers was also evaluated. The in vitro antifungal assays proved that 10 MgHApCh composite layers presented a strong antifungal effect, correlated with the irradiation dose and incubation time. The study of the stability of the 10 MgHApCh gel allowed us to achieve uniform and homogeneous layers that could be used in different biomedical applications.

## 1. Introduction

Gamma radiation is one of the most currently used sterilization methods in the medical field [1]. This method possesses many major advantages, such as improved sterilization safety and higher penetration, compared to other methods of sterilization [2]. On the other hand, the sterilization of biomaterials (such as bone allografts, implants, skin grafts, etc.) could change the final morpho-functional characteristics in a dose-dependent manner [1,3,4]. In previous studies it was emphasized that the use of higher doses of gamma irradiation induces important changes on the morphological, structural, and biological properties of the sterilized materials and exhibits a potent microbicidal effect [1,3,4,5,6,7].

Recently, an increased demand for the provision of new materials for bone substitutes was observed because of the exponential increase in postoperative bone infections, bone trauma, degenerative diseases, and tumors [8]. Thus, bone tissue is one of the most replaced tissues in the human body [9]. Bone tissue has two major components: one represented by an inorganic component, namely hydroxyapatite (Ca_10_(PO_4_)_6_(OH)_2_; HAp), and an organic part mainly represented by the collagen matrix [9].

Synthetic HAp possesses valuable physico-chemical and biological properties (e.g., biocompatibility, osteoconductivity, etc.) that make it suitable for use in various applications (implantology, dentistry, etc.) in the medical field [8,9,10]. To improve the properties of hydroxyapatite and provide it with antimicrobial activity, we chose to dope HAp with magnesium (Mg^2+^). Traces of magnesium are naturally present in bone tissue, dentine, and enamel [11,12,13]. Magnesium has an extremely important role in the muscle, nervous, and skeletal systems’ activity [13,14].

Previous studies have proved that the presence of a chitosan (Ch) matrix, a natural biocompatible biopolymer, could improve the bone regeneration process [15]. If we take into consideration the remarkable properties of Ch and Mg, we could emphasize that coatings of chitosan-coated magnesium-doped hydroxyapatite could represent a good candidate for use in implantology/dentistry. It is well known that almost 90% of fungal infections are determined by *Candida albicans* (*C. albicans*). On the other hand, the study conducted by D. Predoi and collaborators showed that magnesium-doped hydroxyapatite samples exhibit antimicrobial activity against *Pseudomonas aeruginosa* ATCC 27853, *Staphylococcus aureus* ATCC 25923, and *Candida albicans* ATCC 90029 [16].

Hosny A.E.D.M. et al. [17] have highlighted that gamma radiation enhances the antimicrobial activity of zinc oxide nanoparticles against various bacterial strains (*Escherichia coli* (ATCC 25922), *Staphylococcus aureus* (ATCC 25923), *Candida albicans* (ATCC 90028), etc.). In addition, in previous studies it was highlighted that the use of gamma irradiation together with antimycotic drugs may inhibit the development of *Candida albicans* fungal cells [18].

Furthermore, in our previous studies we have reported valuable information regarding the influence of gamma irradiation on the physico-chemical and biological properties of magnesium-doped hydroxyapatite/chitosan composite thin films (x_Mg_ = 0.025 and 0.1) developed by radiofrequency (RF) magnetron sputtering [19] and vacuum deposition [20] techniques. Our findings showed that the radiation dose and magnesium concentration (from the samples) play a crucial role in the final morphological, molecular, chemical compositional, and biological properties of the coatings [19,20].

In this paper, we report for the first time the effect of gamma irradiation on the 10 MgHApCh composite thin films obtained by the spin-coating procedure. The gamma irradiation of the 10 MgHApCh composite layer obtained by spin-coating improved the phyico-chemical and antifungal properties. The 10 MgHApCh composite gel used to prepare the layers was evaluated for its stability by ultrasound measurements. The physico-chemical investigations of 10 MgHApCh composite non-irradiated and irradiated layers such as energy-dispersive X-ray spectroscopy (EDX), scanning electron microscopy (SEM), Fourier-transform infrared spectroscopy (FTIR), atomic force microscopy (AFM), and X-ray photoelectron spectroscopy (XPS) were achieved. The in vitro antifungal activity of the 10 MgHApCh layers was assessed using the reference *Candida albicans* ATCC 10,231 fungal strain.

## 2. Materials and Methods

### 2.1. Materials

The precursors used in order to obtain chitosan-coated magnesium-doped hydroxyapatite (Ca_10−x_Mg_x_(PO_4_)_6_(OH)_2_; x_Mg_ = 0.1; 10 MgHApCh) via sol-gel method were: magnesium nitrate hexahydrate (Mg(NO_3_)_2_·6H_2_O; 99.97 %; Alpha Aesar, Kandel, Germany), calcium nitrate tetrahydrate (Ca(NO_3_)_2_∙4H_2_O, ≥99.0 %, Sigma Aldrich, St. Louis, MO, USA), and diammonium hydrogen phosphate ((NH_4_)_2_HPO_4_; ≥99.0 %, Sigma Aldrich, St. Louis, MO, USA). The ammonium hydroxide (NH_4_OH, 25% NH_3_ in H_2_O (T)), chitosan (C_6_H_11_NO_4_), and ethanol absolute (C_2_H_5_OH) purchased from Sigma Aldrich (St. Louis, MO, USA) were also used. Double distilled water was also used for the synthesis of 10 MgHApCh.

### 2.2. Synthesis of Chitosan Coated Magnesium-Doped Hydroxyapatite (10 MgHApCh)

The synthesis of 10 MgHApCh was conducted in concordance with our earlier studies [16,21,22]. The Ca/P molar ratio was set at 1.67 and the pH value during the synthesis was 11. The synthesis was conducted under continuous stirring at 100 °C for 4 h in air. The synthesis was conducted at room temperature. A hotplate equipped with a magnetic stirrer was also used. The resulting suspension was centrifuged and redispersed in a 2% chitosan solution (under continuous stirring for 4 h at 100 °C). Therefore, the resulting 10 MgHApCh gel was ultrasonically mixed for 6 h in ambient conditions. Finally, the 10 MgHApCh gel suspension was utilized to develop the 10 MgHApCh coatings.

### 2.3. Development of 10 MgHApCh Coatings by Spin-Coating Process

The spin-coating process was used to obtain 10 MgHApCh coatings. Si wafer (Siegert Wafer GmbH, Aachen, Germany) was used as a substrate. To obtain the coating, 0.5 mL of 10 MgHApCh gel suspension was used. The 0.5 mL suspension was dripped with a syringe on top of the Si substrate (polished side). The spin time used was 90 s at a speed of 2000 rpm. The Si substrate was repeatedly coated 3 times. After each coating, the substrate was dried in an oven for 15 min at 100 °C. After the last coating, the final obtained layers were annealed for 2 h at 500 °C. This last treatment aimed to remove the solvents and to obtain a layer with crystalline structure. The obtained 10 MgHApCh composite layers were assigned to three groups with irradiation doses of 0 (10 MgHApCh), 3 Gy (10 MgHApCh-3), and 6 Gy (10 MgHApCh-6) of gamma (γ) irradiation.

### 2.4. Physico-Chemical Characterisations

The resulting 10 MgHApCh gel suspension used to prepare the coatings was investigated by ultrasound measurements to evaluate the stabilization. For the ultrasound measurements, the 10 MgHApCh gel suspension was stirred for 10 min in ambient conditions. The ultrasound measurements of the 10 MgHApCh gel suspension were conducted in agreement with previous studies [23,24].

For the X-ray diffraction (XRD) measurements, a Bruker D8 Advance diffractometer (Billerica, MA, USA) with Cu Kα (Å) radiation was used. The XRD diffractograms were recorded in the 2θ range from 20° to 40° with a step of 0.02°.

The SEM investigations were conducted with the aid of a Hitachi S4500 scanning electron microscope (Hitachi, Tokyo, Japan). An EDX detection system was attached to the microscope to carry out the elemental composition studies of the coatings. SEM and EDX studies were performed without the surface of the irradiated and unirradiated 10 MgHApCh layers being sprayed in vacuum with silver or gold.

The AFM was used to obtain a supplementary acquaintance concerning the surface morphology and roughness of non-irradiated and irradiated 10 MgHApCh composite layers. For this purpose, a microscope NT-MDT NTEGRA Probe Nano Laboratory instrument (NT-MDT, Moscow, Russia) was used. The AFM instrument was operated in semi-contact mode at room temperature and in atmospheric conditions and equipped with a silicon NT-MDT NSG01 cantilever (NT-MDT, Moscow, Russia). The atomic force microscopy (AFM) topographies of non-irradiated and irradiated 10 MgHApCh composite layers were recorded for a surface area of 5 × 5 µm^2^. The R_RMS_ roughness parameter was determined for the non-irradiated and irradiated 10 MgHApCh composite layers from three zones and presented as mean ± SD. The obtained data has been processed with Gwyddion 2.59 software (Department of Nanometrology, Czech Metrology Institute, Brno, Czech Republic) [25].

ATR-FTIR spectroscopy was used to observe the molecular structure of 10 MgHApCh composite layers. For this purpose, a Perkin Elmer SP-100 spectrometer was also employed (Waltham, MA, USA). The ATR-FTIR studies have been conducted in the 450–4000 cm^−1^ spectral range. To obtain the spectra’s second derivative, the protocol described in [26] was used.

The XPS examination was accomplished with the aid of an SES 2002 instrument (Scienta Omicron). The instrument used a monochromatic Al K(alpha) (hν = 1486.6 eV) X-ray source (Scienta Omicron, 18.7 mA, 13.02 kV). The experiments and scans’ analyses were performed in agreement with anterior studies [27,28]. The Casa XPS 2.3.14 software (Shirley background type) [29] and XPS tables [30,31] were used for the analysis of the obtained XPS data. All the values obtained for the binding energy (BE) reported in this paper were charge corrected to C 1 s at 284.8 eV.

### 2.5. Biological Evaluation

The in vitro antifungal properties of the 10 MgHApCh layers unirradiated and irradiated were assessed using the reference Candida albicans ATCC 10,231 (American Type Culture Collection, Manassas, Virginia, United States) fungal strain. The studies were performed according to [32] and the antifungal activity of the composite layers was determined after 24, 48, and 72 h of incubation with the fungal suspensions. The quantitative determination of the fungal cell’s survival was assessed after 24, 48, and 72 h using an adapted method described in detail in [32]. For this purpose, *C. albicans* fungal suspensions with a known fungal density (~5 × 10^6^ colony forming units (CFU)/mL) were obtained from 24 h cultures. The 10 MgHApCh composite layers and Si discs were incubated at 37 °C for 24, 48, and 72 h with the as-obtained fungal suspensions and the temporal dynamics of the fungal cells’ development were evaluated. In this case, for each of the tested incubation periods (24, 48, and 72 h), the fungal suspensions were collected and then incubated on LB agar medium. As a positive control (C+), a free microbial fungal suspension was used. The number of colony forming units per milliliter (CFU/mL) was determined and presented graphically as logCFU/mL function of time. The experiments were performed in triplicate, and the results were expressed as mean ± standard deviation (SD).

The qualitative evaluation of the fungal cells’ adherence and proliferation on the surface of the composite layers was carried out by AFM and confocal laser scanning microscopy (CLSM) studies. For the qualitative assays, fungal suspensions of *C. albicans* were grown on the surface of the 10 MgHApCh composite layers as well as on Si discs immersed in liquid yeast peptone glucose (YPG). Afterwards, the composite thin films were removed from the culture medium for each tested incubation period, washed with sterile saline solution for the removal of unattached fungal cells, and fixed with the aid of cold methanol and prepared for visualization.

The AFM and CLSM studies were performed on the 10 MgHApCh layers exposed for 24, 48, and 72 h to the fungal suspensions. Before the CLSM visualization, the coatings were stained with propidium iodide (PI) in the dark. The CLSM studies were performed using a Leica TCS-SP confocal microscope (Wetzlar, Germany) equipped with a PL FLUOTAR (40_ NA 0.7) objective and operated in reflection and fluorescence modes. In addition, for the CLSM studies, an Ar ion laser (488 nm) was used [33]. ImageJ software (Image J 1.51j8) was used to estimate the dimension of adherent fungal cells [34]. Furthermore, AFM topographies 25 × 25 µm^2^ of the 10 MgHApCh layers exposed to the fungal suspensions were recorded. The experiments were carried out in triplicate and the results of the quantitative assays were depicted as mean ± standard deviation (SD).

## 3. Results

In applications such as orthopedic prosthesis coatings, the uniformity and homogeneity of the coating layer play an important role both in the process of bone recovery and in the prevention of postoperative infections.

**Figure 1:**
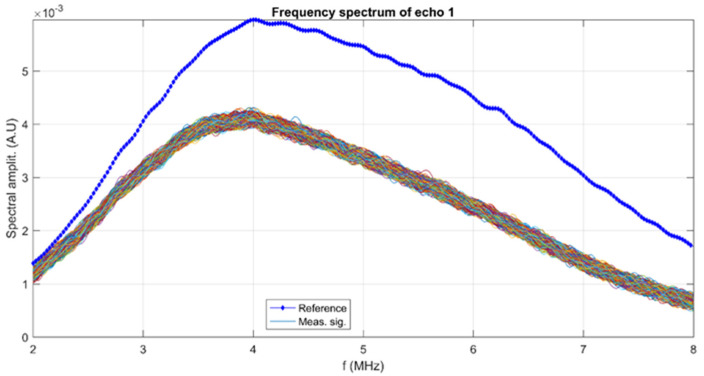
Frequency spectrums for the tested sample (superposing all spectrums) and the reference liquid.

The uniformity and homogeneity of the obtained layers is given by the stability of the 10 MgHApCh composite gel. To evaluate this stability, the 10 MgHApCh composite gel was stirred at 700 rpm for 15 min. In the meantime, the reference liquid, which was double distilled water, was ultrasonically tested using a selected set of experimental parameters, such as the pulse energy, repetition rate, signal amplification, and frequency bandwidth. The reference signals were recorded for processing. The ultrasonic transducers used in this experiment have a central frequency of 5 MHz. The sample was transferred in the same cubic box with coaxial ultrasonic transducers distanced by 20 mm. The ultrasonic signals were recorded every 5 s for later processing. The 1000 recorded ultrasonic signals were analyzed [35]. The signals vs. time graph shows constant amplitudes during the test duration. Consequently, a more detailed analysis was based on computing their frequency spectrums, which were compared with the equivalent spectrum of the reference liquid. The frequency spectrums of the reference liquid (blue dots) and of the 1000 signals of the tested sample are shown in Figure 1. It is apparent that the 1000 spectrums are very close even after 5000 s, the duration of the experiment. This is one indication of the sample’s stability. 

**Figure 2:**
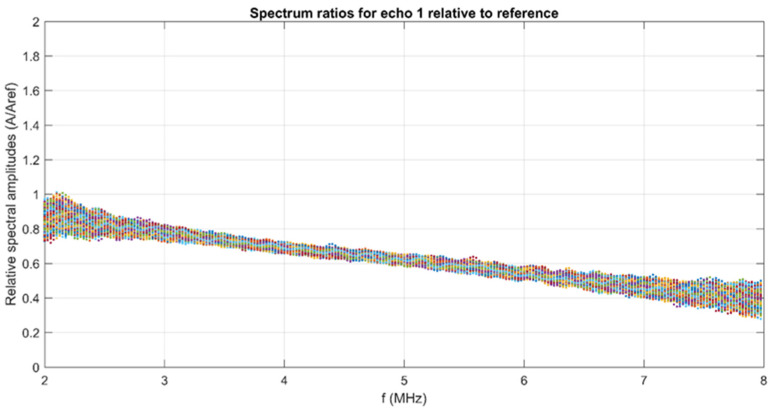
Relative spectral amplitudes of the tested samples.

The frequency spectra can be processed and represented as in Figure 2 as amplitudes ratios in the selected frequency range for all the 1000 records. A linear decrease in this ratio denotes a typical behavior for highly attenuating samples, for which the amplitudes at increasing frequencies are decreasing.

**Figure 3:**
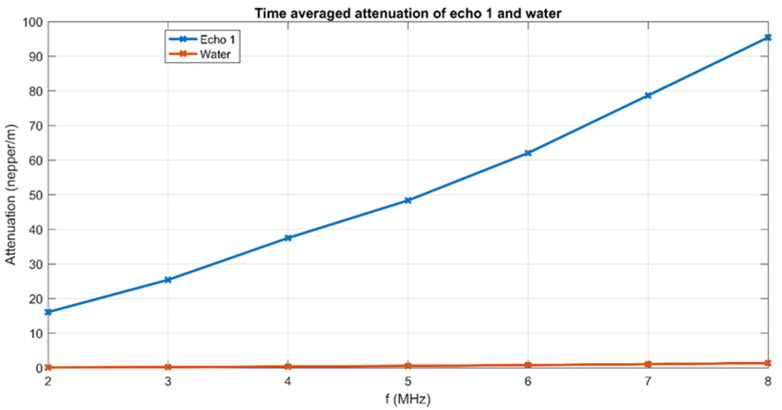
Time averaged attenuation for the 1000 samples in the selected frequency range.

Based on known ultrasonic signals’ attenuation in water, the determination of the attenuation of the tested samples was performed, measured in nepper/m, and the results are shown in Figure 3. The high attenuation of the signals is specific to the high concentration of particles in suspension.

A conclusion of this analysis is that the tested samples are extremely stable (stability parameter $\;s=\frac{1}{<A>}\left|\frac{dA\left(t\right)}{dt}\right|$ = 2.24447 × 10^−6^ s^−1^ in which <*A*> is the time averaged maximal amplitude of the samples, and the time derivative of the recorded amplitudes.

**Figure 4:**
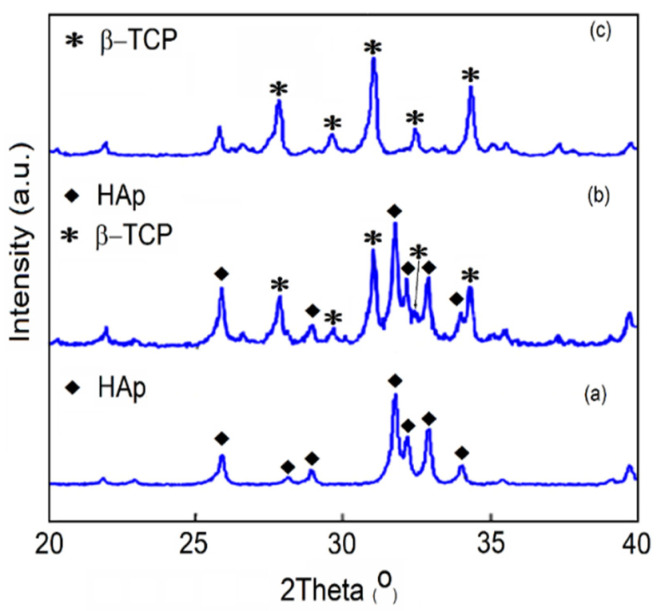
XRD patterns of 10 MgHApCh-0 (**a**), 10 MgHApCh-3 (**b**) and 10 MgHApCh-6 (**c**) samples.

The XRD patterns for the 10 MgHApCh-0, 10 MgHApCh-3 and 10 MgHApCh-6 samples revealed the transformation of HAp to β-tricalcium phosphate (β-TCP) with the increase in the irradiation dose. The transformation of HAp to β-TCP was presented in Figure 4. For the 10 MgHApCh-3 (Figure 4b) sample, the β-TCP phase was dominant. For the 10 MgHApCh-6 (Figure 4c) sample, the X-ray pattern exhibited the well-formed β-TCP phase with an important change in the intensity of the peaks associated with the HAp phase.

**Figure 5:**
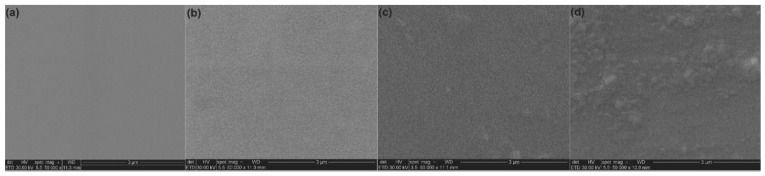
SEM images obtained on Si (**a**), 10 MgHApCh-0 (**b**), 10 MgHApCh-3 (**c**), and 10 MgHApCh-6 (**d**) thin films.

**Figure 6:**
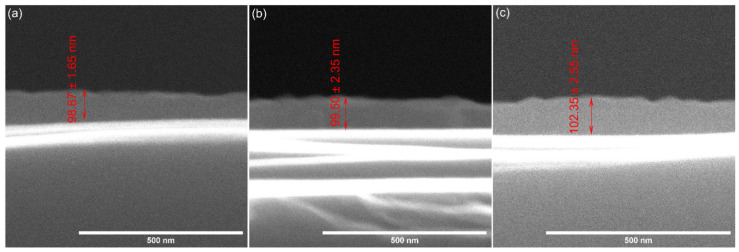
SEM images of transversal cross section of 10 MgHApCh-0 (**a**), 10 MgHApCh-3 (**b**), and 10 MgHApCh-6 (**c**) thin films.

**Figure 7:**
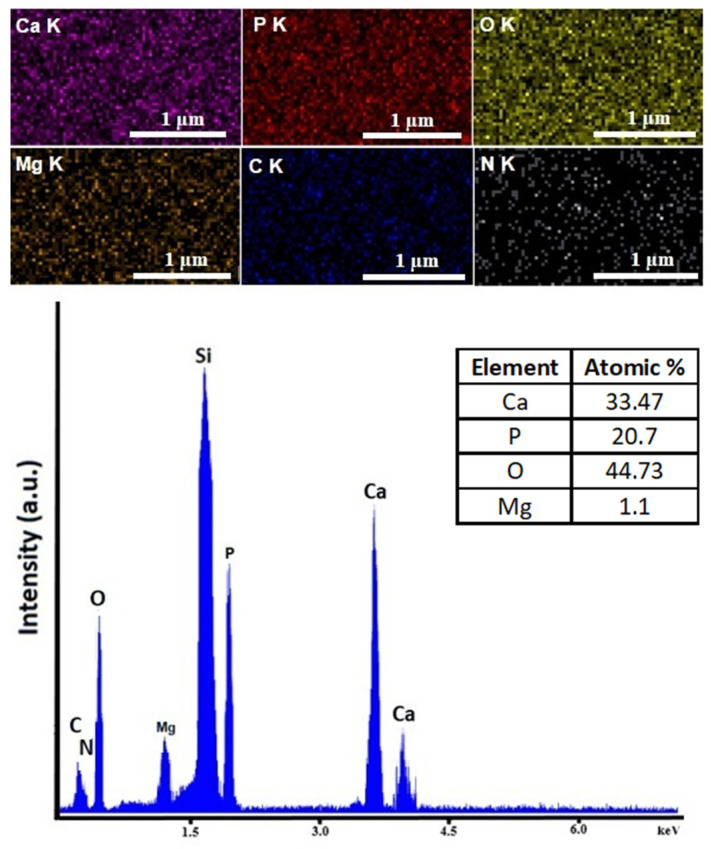
Elemental distribution cartographies of constituent chemical elements along with EDX spectra of 10 MgHApCh-0 thin films.

**Figure 8:**
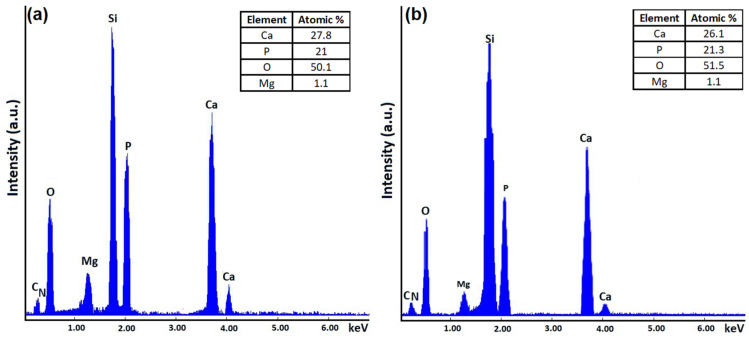
EDX spectra of 10 MgHApCh-3 (**a**) and 10 MgHApCh-6 (**b**) thin films.

Using SEM measurements and an EDX analysis, the morphology and chemical composition of the 10 MgHApCh-0, 10 MgHApCh-3, and 10 MgHApCh-6 composite layers were studied. The results of the SEM and EDX studies are presented in Figure 5, Figure 6, Figure 7 and Figure 8. Firstly, we analyzed the surface morphology of the obtained samples using the spin coating technique. The obtained SEM images emphasize that the thin films’ surface is slightly modified by the increase in the irradiation dose.

As shown in Figure 5, it was noticed that when the irradiation dose increased, the surface became more nanostructured. In the case of an unirradiated sample (Figure 5b), when a smooth surface is highlighted, compared to the case of the samples irradiated at 6Gy (Figure 5d), a structured surface was observed. Furthermore, the surfaces of the studied samples did not show any other defects (e.g., cracks, etc.). Our results are in concordance with the results presented in previous studies reported in the literature [19,20].

Additionally, the results emphasized that the 10 MgHApCh thin films’ surfaces underwent a slight morphological modification after their exposure to irradiation. This behavior could be due to the technique used for obtaining the thin films. On the other hand, we believe that the stability of the gels also played an important role. The good stability of the gel allowed us to obtain more uniform surfaces, preventing the formation of particle agglomerations in the deposition process. Our results indicated that the final dispersion of the final material in a 2% chitosan solution enabled the attainment of more stable layers under irradiation.

The 10 MgHApCh thin films’ thickness was evaluated by SEM studies. Thus, the SEM images of the transversal cross section of the studied thin films are presented in Figure 6. The obtained results show that for the 10 MgHApCh-0 layers, the thickness was around 98 nm, while for the MgHApCh-3 thin films, the obtained thickness was about 99 nm. Furthermore, for the MgHApCh-6 layers, the thickness was around 102 nm. The results obtained for the thickness of the layers were presented as an average ± SD. The results were in accordance with the AFM studies and suggested that the irradiation influences the surface of the coatings. In the cross-section, the data highlighted that the thickness of the layers increased with the increase in the gamma irradiation dose. Moreover, the standard deviation of the thickness obtained from the cross-section measurements also increased for the irradiated samples, which could be explained by an increase in the surface roughness, which was also obtained from the AFM topographies.

The results of the EDX studies regarding the chemical composition of the samples are presented in Figure 7 and Figure 8. Additionally, the elemental distribution maps of the main chemical constituents of the 10 MgHApCh-0 sample are presented (Figure 7).

The EDX spectra revealed the presence of phosphorus (P), nitrogen (N), calcium (Ca), magnesium (Mg), oxygen (O), and carbon (C). All these chemical elements are specific to the chitosan-coated magnesium-doped hydroxyapatite sample’s composition. Moreover, the presence of the silicon (Si) line is attributed to the substrate. Therefore, the presence of other additional lines specific to the impurities were not observed in the obtained EDX spectra. Similar results were obtained for the other irradiated samples. Additionally, the elemental distribution cartographies are presented in Figure 7. Our results highlight the good homogeneity and distribution of the most important chemical elements in the 10 MgHApCh-0 thin films (Figure 7).

**Figure 9:**
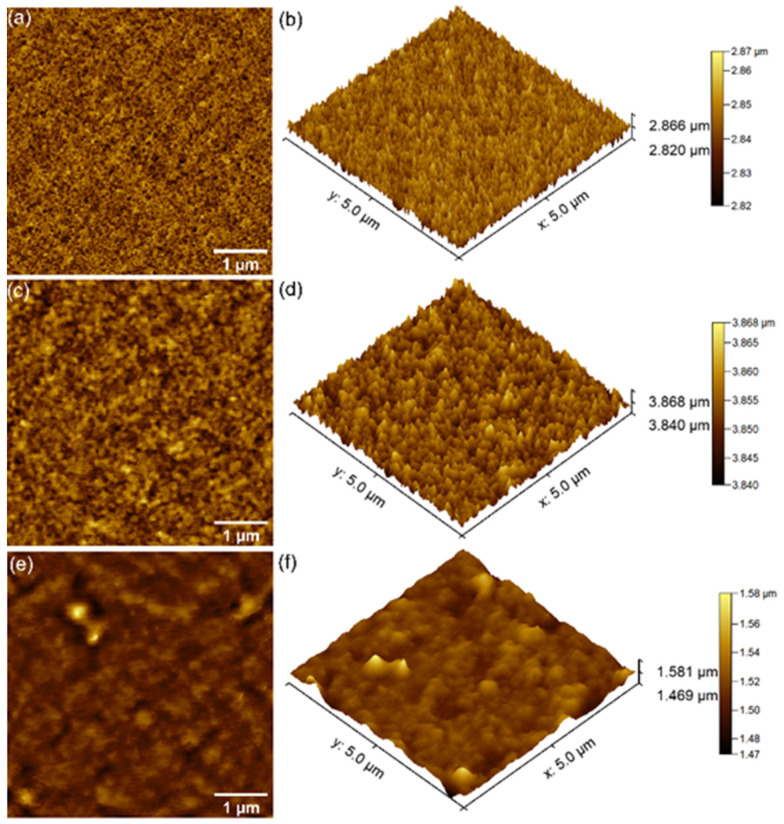
2D AFM topographies of 10 MgHApCh-0 (**a**), 10 MgHApCh-3 (**c**) and 10 MgHApCh-6 (**e**) composite layers and their 3D representations (**b**,**d**,**f**).

The surface topography of the chitosan-coated magnesium-doped hydroxyapatite (10 MgHApCh) composite layers before and after irradiation with different doses of gamma rays was investigated with AFM. For this purpose, the composite layers were scanned in ambient conditions on a surface of 5 × 5 µm^2^. The 2D AFM surface topographies and the 3D portrayal of the nonirradiated and irradiated 10 MgHApCh composite layers are presented in Figure 9a–f. The results obtained from the AFM scanning showed that the surface topographies of the 10 MgHApCh layers unirradiated and irradiated with irradiation doses of 0, 3, and 6 Gy of gamma irradiation were similar, and that the gamma irradiation did not produce any major effects on the coating’s surface. The 2D AFM images recorded on a surface of 5 × 5 µm^2^ revealed the presence of a homogenous, continuous, and well-structured layer for all the investigated samples. Moreover, the AFM studies highlighted that there were no visible fissures or cracks or any other undesired forms of unevenness on the surface of the unirradiated and irradiated 10 MgHApCh composite layers. Supplementary information about the surface topographies of the 10 MgHApCh composite layers were also obtained from the 3D depiction of the AFM data. The 3D images of the unirradiated and irradiated 10 MgHApCh composite layers revealed that no noticeable discontinuities or unevenness were observed on the investigated surfaces. Moreover, the AFM data also suggested that the increase in the irradiation dose resulted in a slight increase in the porosity of the samples. In addition, the root mean square (R_RMS_) parameter values were determined from the AFM data for all the investigated samples. The value for the R_RMS_ parameter 10 MgHApCh-0 was determined at 3.85 ± 1.54 nm, while the values for the R_RMS_ parameters for 10 MgHApCh-3 and 10 MgHApCh-6 were determined at 6.75 ± 1.25 and 9.75 ± 1.77 nm. The AFM studies highlighted that the surface roughness of the samples increased with the irradiation dosage. In addition, the AFM results were in concordance with the data obtained by the SEM measurements.

**Figure 10:**
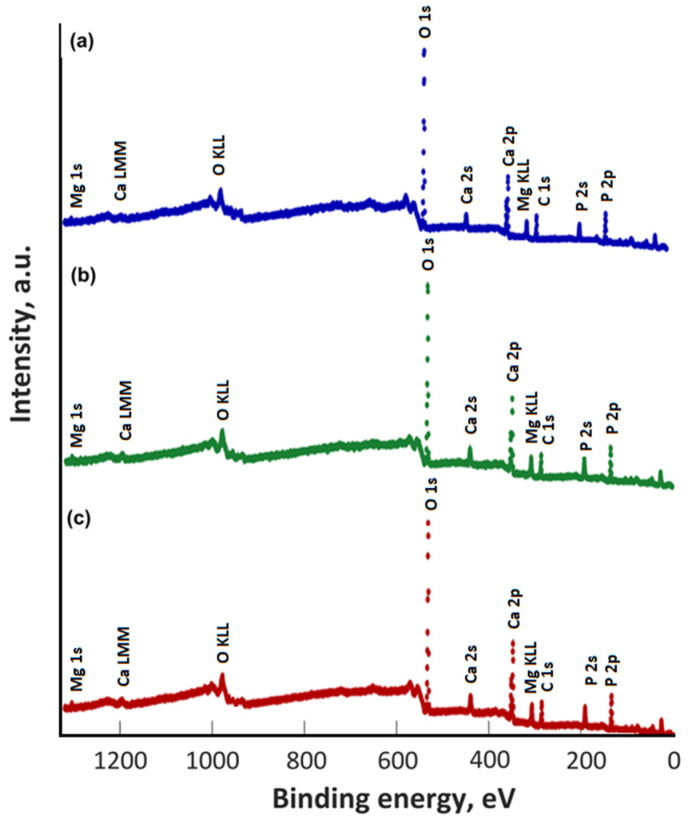
XPS survey results of 10 MgHApCh-0 (**a**), 10 MgHApCh-3 (**b**), and 10 MgHApCh-6 (**c**).

The XPS studies of the 10 MgHApCh layers unirradiated and irradiated with different irradiation doses of 0, 3, and 6 Gy of gamma irradiation were achieved. Thus, the presence of magnesium ions in the unirradiated and irradiated samples of the 10 MgHApCh layers was highlighted. Figure 10 shows the general XPS spectra of the unirradiated and irradiated 10 MgHApCh thin films. The constituent elements were present in the general XPS spectrum of all the 10 MgHApCh samples (unirradiated and irradiated).

**Figure 11:**
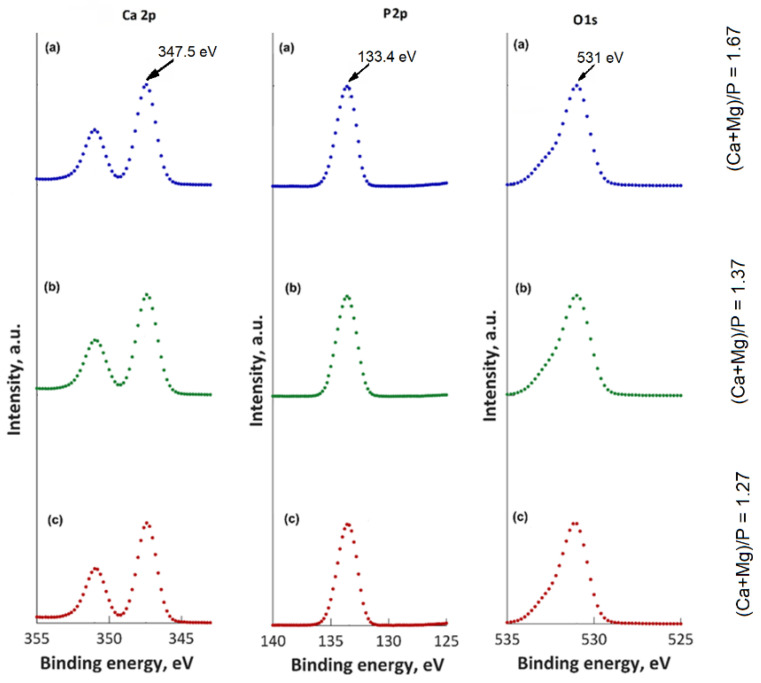
High-resolution XPS spectra for Ca 2p, P2p, and O 1 s spectra of 10 MgHApCh-0 (**a**), 10 MgHApCh-3 (**b**), and 10 MgHApCh-6 (**c**) layers.

Figure 11 depicts the high-resolution XPS spectra of P 2p, O 1 s, and Ca 2 p of the unirradiated and irradiated 10 MgHApCh composite layers. The presence of magnesium was highlighted in all the analyzed 10 MgHApCh samples. This result confirms that calcium ions were substituted with magnesium ions during the synthesis process. The principal peak of the O1 s XPS spectra of 10 MgHApCh-0 (Figure 11a) was located at 531.21 eV. The principal peaks of the P 2p and Ca 2p of 10 MgHApCh-0 (Figure 11a) were located at 133.21 eV and 347.31 eV, respectively. The values of the binding energies (BEs) of O 1 s, P2p, and Ca 2p were attributed to HAp in agreement with previous studies [36,37,38]. For the irradiated 10 MgHApCh composite layers (Figure 11b,c), the high-resolution XPS spectra of P 2p, O 1 s, and Ca 2 p exhibited an easy peak shift with the increasing of the irradiation dose. For the 10 MgHApCh-3 (Figure 11b) and 10 MgHApCh-6 (Figure 11c) layers, the peaks’ positions of Ca2p were localized at BEs of 347.52 eV and 347.72 eV, respectively. The peaks’ positions of P2p for the 10 MgHApCh-3 (Figure 11b) and 10 MgHApCh-6 (Figure 11c) layers were located at BEs of 133.4 and 133.63 eV, while the peaks’ positions of O 1 s were identified at BEs of 531.29 and 531.34 eV.

**Figure 12:**
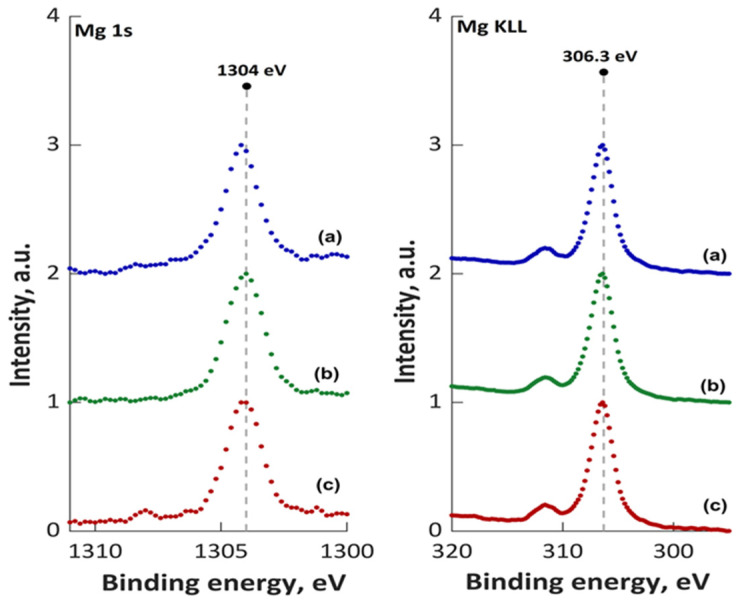
XPS high resolution spectra of Mg 1 s and Mg KLL of 10 MgHApCh-0 (**a**), 10 MgHApCh-3 (**b**), and 10 MgHApCh-6 (**c**) layers.

The XPS high resolution spectra of Mg 1 s and Mg KLL of the unirradiated and irradiated 10 MgHApCh composite layers were also presented (Figure 12). The peak position of Mg 1 s for unirradiated 10 MgHApCh layers (Figure 12a) was detected at a BE of 1302 eV while the peak position of Mg KLL (Figure 12b) was identified at BE of 304.31 eV. Thus, the Mg 1 s peaks’ positions of the irradiated 10 MgHApCh composite layers were located at BEs of 1302.94 (Figure 12b) and 1304.1 (Figure 12c). The Mg KLL peaks’ positions of the irradiated 10 MgHApCh composite layers were located at BEs of 305.12 eV for the MgHApCh-3 composite layers (Figure 12b) and 306.09 eV for the MgHApCh-6 composite layers (Figure 12c).

The XPS studies allowed us to evaluate the ratio of (Ca + Mg)/P. It was observed that that the ratio of (Ca + Mg)/P decreases from 1.67 (for the non-irradiated sample) to 1.27 (for the MgHApCh-6 sample). For the MgHApCh-3 sample, the ratio of (Ca + Mg)/P was 1.37. The data obtained from the XPS studies are in agreement with those obtained from the EDX evaluation of these layers. The XPS studies have shown that the ratio of (Ca + Mg)/P decreases as the irradiation dose increases, which highlights the formation of a calcium-deficient apatite. The decrease in the ratio of (Ca + Mg)/P as the irradiation dose increases was also observed in the EDX analysis. The XPS analysis of the irradiated 10 MgHApCh layers suggested that the magnesium and calcium contained in the surface layer are from β-tricalcium phosphate (β-TCP) and hydroxyapatite (HAp). The present results show that the transformation of HAp to β-TCP depends on the irradiation dose [39,40,41]. This transformation could occur over a wider range for the (Ca + Mg)/P ratio than that presented in previous studies [42,43]. According to previous studies [44,45,46] the transformation of HAp into β-TCP can be influenced by several factors, such as temperature, pH value, substituted ions (it is known that hydroxyapatite is very hospitable and allows for the replacement of the Ca^2+^, PO_4_^3−^, or OH^−^ groups with other ions), and thermal stability. In agreement with Kannan et al. [47], the HPO_2_^4−^ ions plays an important role in the β-TCP formation (Equation (1)):

Ca_10−x_(HPO_4_)_x_(PO_4_)_6−x_(OH)_2−x_ → _1−x_Ca_10_(PO_4_)_6_(OH)_2_ + 3·Ca_3_(PO_4_)_2_ + xH_2_O (1)

**Figure 13:**
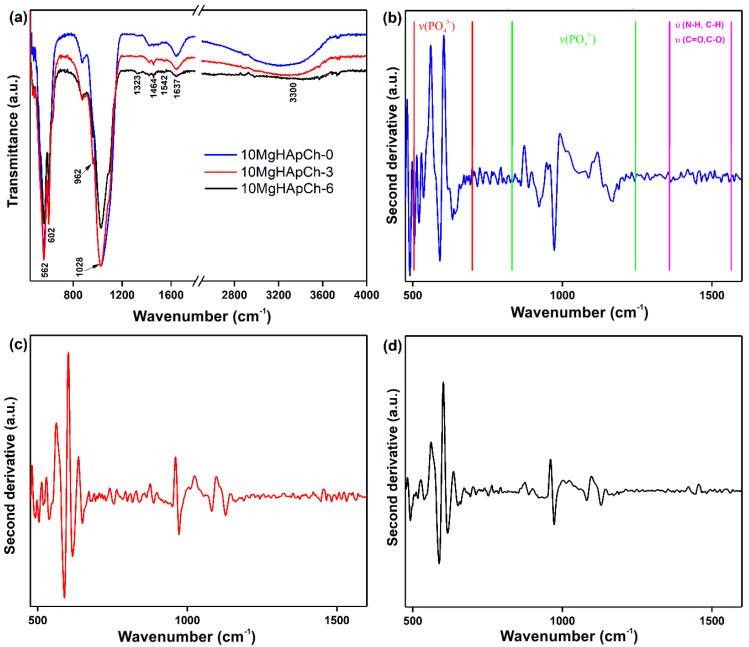
ATR-FTIR spectra of 10 MgHApCh thin films (**a**) and second-derivative spectra obtained for 10 MgHApCh-0 (**b**), 10 MgHApCh-3 (**c**), and 10 MgHApCh-6 (**d**) thin films.

In Figure 13a, the ATR-FTIR spectra obtained for the 10 MgHApCh-0, 10 MgHApCh-3 and 10 MgHApCh-6 thin films are revealed, respectively. In the general spectra (Figure 13a) the main peaks’ characteristics towards the hydroxyl and phosphate groups from the HAp structure along with the peaks that are attributed to the C–H/C–O vibration from the chitosan structure can be observed. All these peaks suggest the formation of a chitosan-coated magnesium-doped hydroxyapatite structure. Thus, the peak found at about 480 cm^−1^ belongs to ν_2_(PO_4_^3−^), while the bands from 562, 602, and 633 cm^−1^ are attributed to ν_4_(PO_4_^3−^) vibration [19,20]. The peak found at 962 cm^−1^ is specific to ν_1_(PO_4_^3−^); meanwhile, the peaks observed at around 1028 and 1094 cm^−1^ belong to the ν_3_(PO_4_^3−^) from the hydroxyapatite structure [19,20]. The presence of chitosan in the studied samples was emphasized by the presence of the peaks in the 1300–1600 cm^−1^ spectral range. In the spectral range mentioned above, we observed the peaks that are characteristic of the C–H/N–H/C=O/C–O vibration from chitosan [20]. The broadened peak observed at ~1542 cm^−1^ could belong to ν (NH_2_) from the chitosan structure [32,48]. In the 2700–3600 cm^−1^ spectral region, the vibrational bands specific to the vibration of the –NH_2_ group from the chitosan structure are overlapped with the vibrational bands of the hydroxyl groups from the hydroxyapatite structure [42]. The obtained ATR-FTIR spectra suggest that as the irradiation dose to which the sample was exposed increases, the peaks’ intensity decreases and their position is slightly displaced. Similar results have been previously reported by B. Bita et al. [19] and D. Predoi et al. [20].

For greater accuracy, the second order derivatives of the FTIR spectra were obtained. The second-derivative curves obtained for the studied thin films are exhibited in Figure 13b–d. Shown in Figure 13b–d, the main peaks that belong to ν_2,_ ν_1,_ ν_3,_ and ν_4_ (PO_4_^3−^) vibration from the hydroxyapatite structure were observed. The mentioned peaks are found in the following spectral region: 460–630 cm^−1^ (for ν_2_ and ν_4)_ and 960–1100 cm^−1^ (for ν_1_ and ν_3_) [19,32]. Another peak that belongs to chitosan’s structure was found at ~989 cm^−1^ and arise due to ν (C–H/C–O) [19]. Moreover, in the 1300–1600 cm^−1^ spectral domain, peaks that belong to the ν (C–H/N–H/C=O/C–O) vibration from chitosan’s structure were noticed. Therefore, the results presented in this paper are in concordance with those reported for similar coatings obtained by the magnetron sputtering technique/vacuum deposition [19,20]. The intensity of the peaks associated with chitosan structure are lower as a result of the thermal treatment that was applied to the layers. This result is in agreement with previously reported studies [49]. The thermal treatment of the 10 MgHApCh enabled the acquirement of well-structured surfaces, and our results are in agreement with previous studies [50] reported in the literature.

*C. albicans* is one of the most common etiological agents of fungal infections, and it has been reported as the fourth most frequent cause of infection in US hospitals [51] as a consequence of the increasing numbers of susceptible/compromised patients [52,53,54]. The most important factor that is responsible for the pathogenesis of candidiasis is biofilm formation. *C. albicans* can develop biofilms on both inert and biological surfaces. In particular, *C. albicans* is the most common pathogen isolated in catheter-related blood stream infections leading to high morbidity and mortality rates [52]. The development of biofilms complicates any course of treatment, as sessile cells within these biofilms can resist conventional drugs [55,56]. In this context, the research and development of new antifungal agents are urgently needed [57].

**Figure 14:**
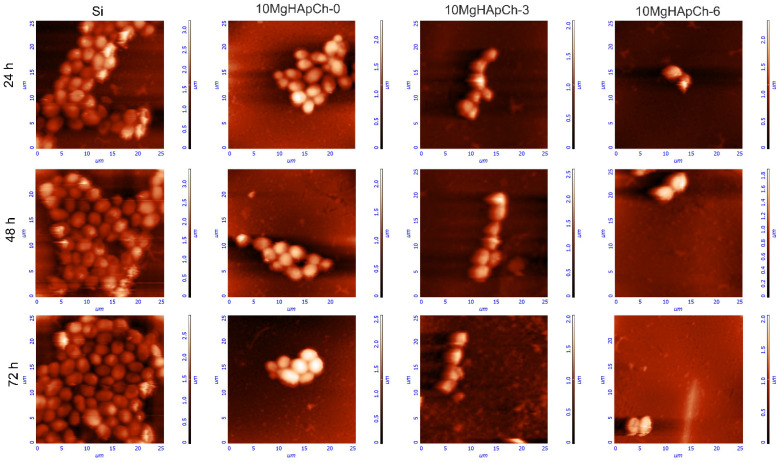
2D AFM surface topography of *Candida albicans* ATCC 10231 cell development on Si discs, 10 MgHApCh-0, 10 MgHAp-Ch-3, and 10 MgHAp-Ch-6 composite layers after 24 h, 48 h, and 72 h of incubation collected on an area of 25 × 25 µm^2^.

**Figure 15:**
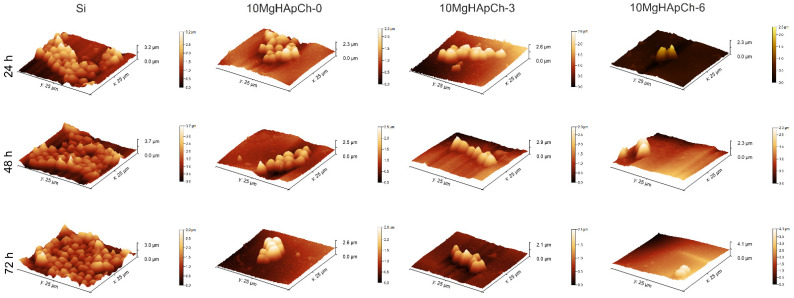
3D representation of AFM surface topography of *Candida albicans* ATCC 10231 cell development on Si discs, 10 MgHApCh-0, 10 MgHAp-Ch-3, and 10 MgHAp-Ch-6 composite layers after 24 h, 48 h, and 72 h of incubation collected on an area of 25 × 25 µm^2^.

Studies regarding the adherence and proliferation of the *C. albicans* fungal cells on the surface of Si discs, 10 MgHApCh-0, 10 MgHApCh-3, and 10 MgHApCh-6 composite layers were performed using AFM. For this purpose, AFM topographies of the Si discs, 10 MgHApCh-0, 10 MgHApCh-3, and 10 MgHApCh-6 composite layers incubated with *C. albicans* fungal cells for three different time intervals, which were recorded on a surface of 25 × 25 µm^2^. The results of the AFM surface topography of the non-irradiated and irradiated 10 MgHAp-Ch composite layers as well as the Si discs incubated with *C. albicans* fungal cells at different time intervals are presented in Figure 14 and Figure 15. The 2D AFM surface topographies depicted in Figure 14 highlighted that the fungal cell development was inhibited by all the 10 MgHAp-Ch composites’ layers for all the tested time intervals. Furthermore, the 2D AFM topographies also showed that the Si disc did not inhibit the *C. albicans* cells’ adherence and development. Moreover, the 2D AFM images suggested that the Si discs’ surfaces allowed for the adherence and proliferation of the fungal cells on their surfaces at all the investigated time intervals. Furthermore, the results of the AFM studies emphasized that the antifungal properties of the 10 MgHAp-Ch composite layers increased with the increase in the incubation time. The 2D AFM images depicted that all the tested samples, except for the Si discs, started inhibiting the *C. albicans* fungal cell development in the first 24 h of incubation and that the antifungal effect of the samples increased significantly with the increase in the incubation time. The 2D AFM images of the samples incubated with the *C. albicans* fungal suspensions for three different time intervals depicted that the morphology of the fungal cells was typical for that of *C. albicans* cells, having round to ovaloid shapes with sizes ranging from 2.14 to 5.75 µm. In addition, the AFM studies also revealed that the fungal cells’ adhesion was inhibited only after 24 h of incubation for all the investigated samples, with the exception of the Si discs. Moreover, the AFM results also confirmed that the 10 MgHApCh composite layers prevented the development of *C. albicans* biofilms on the tested surfaces. Nonetheless, the AFM 2D images indicated that a better antifungal activity was observed in the case of the 10 MgHApCh-6 composite layers. The AFM results suggested that while all the 10 MgHApCh samples inhibited the fungal cells’ adherence and development, a stronger, almost eradicative, inhibitory effect against the *C. albicans* fungal cells was observed in the case of the 10 MgHApCh-3 and 10 MgHApCh-6 composite layers after 72 h. All the 10 MgHApCh samples exhibited a stronger antifungal activity after 72 h of incubation. Moreover, both the 2D AFM images as well as their 3D representations depicted in Figure 15 emphasized that on the surface of the composite layers exposed to *C. albicans* fungal suspension for three different time intervals, the adhered cells were mostly isolated and there was no indication of hyphal formations, which are important in the initial phases of biofilm formation.

Moreover, we reported that the antifungal properties of the samples were increased by irradiation. The SEM and AFM investigations revealed a good homogeneity of the surface of the non-irradiated and irradiated 10 MgHApCh layers.

**Figure 16:**
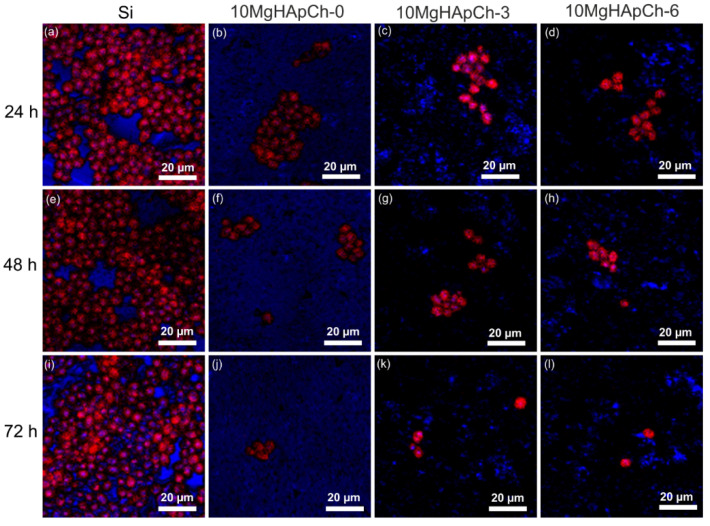
2D CLSM images of *Candida albicans* ATCC 10231 cell development on Si discs (**a**,**e**,**i**), 10 MgHApCh-0 (**b**,**f**,**j**), 10 MgHApCh-3 (**c**,**g**,**k**), and 10 MgHApCh-6 (**d**,**h**,**l**) coatings after 24, 48, and 72 h of incubation.

Additional studies regarding the adherence and development of the *C. albicans* fungal cells on the surface of the 10 MgHApCh composite layers were performed with the aid of the CLSM technique. The CLSM visualization of the *C. albicans* adherence and development on the surface of the nonirradiated and irradiated 10 MhHApCh coatings was conducted for three different time intervals of incubation (24, 48, and 72 h). An Si disc was used as a control for the in vitro qualitative antifungal assays. The results of the CLSM qualitative assays are depicted in Figure 16a–l.

The CLSM visualization studies revealed that the cells attached to the surfaces of the 10 MhHApCh composite layers presented the characteristic morphology of *C. albicans* fungal cells, having an “ovaloid” shape and sizes ranging between 2.55 to 5.85 µm. The CLSM observation highlighted that the 10 MgHApCh composite layers nonirradiated and irradiated with different gamma-irradiation doses were successful in inhibiting *C. albicans* cells’ ability to develop biofilm on their surface. On the other hand, the CLSM images clearly suggested that the Si discs favorized the adherence and the development of *C. albicans* cells on their surface, having a positive influence on their proliferation and their ability to organize themselves in biofilms. These results are in good accordance with the data obtained by the AFM investigations. Moreover, the CLSM visualization showed that the 10 MhHApCh composite coatings were effective in inhibiting the *C. albicans* adherence to their surface in the first 24 h. The CLSM images obtained on surfaces of 85 × 85 µm^2^ undoubtably showed that the 10 MhHApCh composite coatings inhibited the development of *C. albicans* fungal cells for all the tested time periods. Furthermore, the CLSM observation also demonstrated that the cells attached to the surfaces of the 10 MhHApCh coatings were not organized in major conglomerates and that after 48 h they started to become singular and unevenly distributed on their surface. In addition, the CLSM results also suggested that the antifungal effect of the coatings could be correlated with the increase in the incubation time and with the increase in the gamma radiation dose. The results presented in Figure 16a–l show that the adherence and development of the fungal cells considerably decreased with both the increase in the incubation time and the dose of irradiation. The data highlighted that after 72 h, the cells that had adhered to the 10 MgHApCh-6 coating’s surface were almost extinct. These results are also in accordance with the ones suggested by the AFM studies.

**Figure 17:**
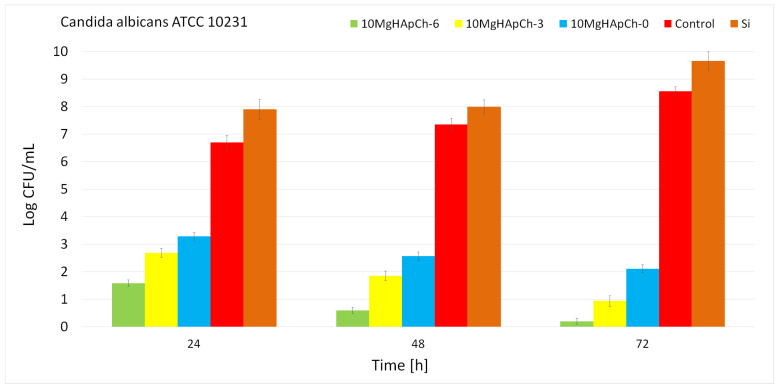
The graphical representation of the antimicrobial activity of Si discs, 10 MgHApCh-0, 10 MgHApCh-3, and 10 MgHApCh-6 composite layers against *C. albicans* (ATCC^®^ 10231) fungal cells after 24, 48, and 72 h of incubation.

The quantitative antifungal evaluation of the 10 MgHApCh composite layers was also performed against *C. albicans* ATCC 10231 fungal strain. The graphical representation of the effects of the 10 MgHAp-Ch composite layers and Si discs on the development of *C. albicans* ATCC 10231 colony-forming units (CFU) at three different time intervals (24 h, 48 h, and 72 h) are depicted in Figure 17.

The results of the quantitative antifungal assays emphasized that the CFU’s development was inhibited early on in the 24 h of incubation in the case of all the 10 MgHAp-Ch composite layers. The in vitro antifungal studies emphasized that there was a significant decrease in the number of fungal colonies in the first 24 h for both the unirradiated and irradiated 10 MgHApCh composite layers compared to the number of colonies formed in the case of the positive control culture (C+). Moreover, the quantitative assays also demonstrated that the Si discs promoted the development of *C. albicans* fungal cells, as shown by the considerable increase in the number of colonies developed on the surface of the Si discs compared to the positive control. Moreover, the results of the quantitative antifungal studies emphasized that the antifungal properties of the samples were influenced by the incubation period. Moreover, the results also suggested that the antifungal properties were more noticeable in the case of the irradiated samples. Furthermore, the data also demonstrated that the irradiation dose influences the antifungal activity, with the 10 MgHApCh-6 exhibiting the strongest antifungal activity. On the other hand, the results of the quantitative antifungal studies showed that the coatings strongly inhibited *C. albicans* fungal cell development and substantially reduced the number of colonies, almost towards extinction, after 72 h of exposure to the fungal suspensions. These results are in good accordance with the qualitative assessment performed using AFM and CLSM techniques and with previously reported studies [19,20,32,58,59,60,61].

The results of the antifungal assays attested that gamma irradiation could be successfully used as an enhancer of antifungal activity in the development of novel antimicrobial devices. An enhancement of the antifungal effect was observed in the case of the irradiated 10 MgHApCh composite layers. This behavior may be due to an increase in the amount of TCP as the irradiation dose increases. The different doses of gamma irradiation exhibit a different impact on the 10 MgHApCh layers, leading to the formation of a calcium-deficient surface layer and to an increase in its porosity. Our studies validate the data available in the literature since high-energy ion beam irradiation has been shown to lead to an improvement of the physico-chemical and biological properties of composite layers based on hydroxyapatite with an increase in porosity upon irradiation [62,63,64]. The presence of Magnesium (Mg) in the chitosan-coated magnesium-doped hydroxyapatite (10 MgHApCh) structure allowed us to obtain more uniform and better-structured layers compared to the layers obtained in previous studies [65]. In addition, the presence of Mg in our samples allowed us to obtain coatings with superior antifungal activity compared to those of chitosan–hydroxyapatite [32]. On the other hand, the new technique used for obtaining Chitosan-coated magnesium-doped hydroxyapatite (MgHApCh) layers allowed us to obtain layers that after irradiation keep their homogeneity without showing cracks on their surface. In addition, as a result of the stable gels used to obtain these layers, it was possible to generate better-structured layers compared to previous studies [19,65]. Moreover, the structural changes in the surface layer after irradiation could also be responsible for the improvement of the biological properties, which would be in agreement with previous studies [66,67]. During the years, various mechanism responsible for the antimicrobial properties of materials have been proposed and reported in different studies [62,68,69,70,71]. The mechanisms based on the interaction between the positive charge of the investigated surfaces of the studied materials and the anionic parts of the microbial cells’ structures that could lead to the disruption and ultimately to the death of the microbial cells is one of the most frequently cited in the literature [71,72,73,74]. In the case of chitosan samples and chitosan-based materials, the main antimicrobial mechanism is based on the interactions that take place between the cytoplasmic membrane of the microbial cells and chitosan. In this case, the cell permeability is being modified and the transport of nutrients to the microbial cell is interrupted, eventually resulting in the microbial cells’ death [71,72,73,74]. A less common and unelucidated mechanism is represented by the binding of chitosan constituents to the DNA strand, which could lead to the condensation of chromatin and a disruption of the synthesis of essential proteins and enzymes that ultimately results in cell lysis and death. Another mechanism explaining chitosan’s antimicrobial properties is based on its metal ions’ chelating properties [73,74,75].

Furthermore, it has been reported that the antimicrobial properties of materials and coatings are usually also strongly influenced by various parameters, such as the type of microbial cells, the type of the antimicrobial agent’s phase (powder, dispersion, nanoparticle, coatings, etc.), and other various factors such as the temperature at which the experiments are conducted, the pH value, etc. In the case of composite coatings, the antifungal properties could also be influenced by the interactions that appear between the substrate and the different layers. In our study, the results of the qualitative and quantitative in vitro antifungal assays suggested that the antifungal activity presented by the 10 MgHAp-Ch composite layers could be attributed to the presence of chitosan and magnesium ions, while the Si discs did not present any inhibitory effects against the *C. albicans* fungal cells’ attachment and growth. Moreover, the data also revealed that both the incubation time and the irradiation dose improved the antifungal properties of the composite layers. This behavior could be attributed either to the synergistic interaction between the substrate and the coating material or to the physico-chemical effects produced by the irradiation dose. Nonetheless, the exact mechanisms of the antimicrobial activities of these materials and coatings in general are still not completely elucidated and thereby require future complex studies to generate a better understanding of the involved antimicrobial mechanisms.

These things considered, the results presented in this study attested that the 10 MgHApCh composite layers prepared by the spin-coating procedure and irradiated with different gamma radiation doses may be considered favorable candidates for the future development of novel antifungal biomedical devices.

## 4. Conclusions

The effect of gamma irradiation on 10 MgHApCh composite layers prepared by spin-coating procedure was analyzed for the first time. The gamma irradiation of the 10 MgHApCh composite layers obtained by spin-coating improved their physico-chemical and antifungal properties. The XPS results were in good conformity with the EDX evaluation and revealed that the calcium decreases when the irradiation doses increase. The SEM and AFM investigations revealed the good homogeneity of the surface of the non-irradiated and irradiated layers. The amount of TCP in the surface layer increased with the irradiation dose. In addition, the layers became amorphous when the irradiation dose increased. Furthermore, there was an enhancement of their antifungal effect upon irradiation, which would help improve the bone cell attachment of the biomaterials used in dental and orthopedic implants. Following these studies, we can say that the 10 MgHApCh non-irradiated and irradiated layers could be used in various biomedical applications to prevent the post-surgical infections of dental and orthopedic devices.

## Data Availability

Not applicable.
